# Preoperative CT Suspicion of Main Pancreatic Duct Injury in Isolated Blunt Pancreatic Trauma: A Case Report With Operative Correlation

**DOI:** 10.1002/ccr3.73760

**Published:** 2026-10-17

**Authors:** Syeda Sarah Kazmi, Syeda Zubaria Qamar, Ahmed Ali, Manahil Sohail Fatani, Syed Ali Haider, Muhammad Farhan, Ahmed Asad Raza, Abedin Samadi

**Affiliations:** ^1^ Dow University of Health Sciences Karachi Pakistan; ^2^ Department of Medicine Karachi Medical and Dental College Karachi Pakistan; ^3^ Jinnah Postgraduate Medical Centre Karachi Pakistan; ^4^ Kabul University of Medical Sciences Abu Ali Sina Kabul Afghanistan

**Keywords:** AAST Grade III, blunt abdominal trauma, case report, computed tomography, distal pancreatectomy, main pancreatic duct injury, pancreatic trauma

## Abstract

An 18‐year‐old male had persistent tachycardia after blunt abdominal trauma. CT signs raised suspicion of main pancreatic duct disruption in the tail (suspected AAST Grade III). Exploratory laparotomy confirmed ductal transection, managed by spleen‐preserving distal pancreatectomy. Six‐month follow‐up showed no fistula or clinical pancreatic insufficiency.

## Key Clinical Message

In blunt abdominal trauma with persistent tachycardia despite resuscitation, isolated pancreatic injury should be suspected even without external signs. Indirect signs on contrast‐enhanced CT, such as focal parenchymal discontinuity with a peripancreatic collection, should raise suspicion of main pancreatic duct disruption, although CT alone is not definitive. In a stable patient, this suspicion can support timely operative planning; here, spleen‐preserving distal pancreatectomy was followed by an uncomplicated recovery with no pancreatic fistula or clinical evidence of pancreatic insufficiency at 6 months.

## 1 Introduction

Pancreatic injuries account for a small proportion of abdominal trauma cases and are frequently associated with injuries to other abdominal organs [1]. Isolated pancreatic trauma is rare and diagnostically challenging due to the retroperitoneal location of the pancreas and the often subtle early clinical signs [2]. The integrity of the main pancreatic duct is the most important determinant of management and prognosis [3]. We present a case of isolated Grade III pancreatic injury managed with spleen‐preserving distal pancreatectomy. The management described reflects established guideline‐based care [1], and the case is not presented as a novel entity. Its value is educational: it correlates each indirect CT sign on which preoperative suspicion of ductal injury rested with the operative findings, and it illustrates how such a suspicion, together with persistent tachycardia despite a relatively benign abdominal examination, supported timely operative planning in a haemodynamically stable patient in whom dedicated ductal imaging could not be obtained without delaying surgery.

## 2 Case Presentation

### 2.1 Patient Information

An 18‐year‐old male presented to the emergency department following a road traffic accident sustained as a pillion passenger on a motorcycle. The mechanism of injury involved blunt abdominal trauma resulting from a posterior impact during the collision. All identifying details have been withheld, and the patient is described only by age, sex, and mechanism of injury.

His primary presenting concerns were abdominal pain and persistent tachycardia that did not resolve with initial fluid resuscitation. No vomiting, haematemesis, haematuria, or loss of consciousness was documented at presentation.

No significant past medical or surgical history was documented. He was not taking regular medication and had no known drug allergies. There was no family history of pancreatic or hereditary metabolic disease, and no relevant genetic information was available. He was a student with no reported tobacco, alcohol, or substance use, and no psychosocial factors were identified that influenced his presentation or management.

Relevant past interventions and their outcomes: the patient received prehospital and emergency department resuscitation according to Advanced Trauma Life Support principles, including intravenous crystalloid infusion. Tachycardia persisted despite this intervention, which prompted urgent cross‐sectional imaging.

### 2.2 Clinical Findings

On arrival, the patient was conscious, fully oriented and maintaining his own airway, with a Glasgow Coma Scale of 15. He remained tachycardic despite initial fluid resuscitation, although systolic blood pressure was maintained, and he was otherwise haemodynamically stable, which permitted cross‐sectional imaging to be undertaken. He was afebrile, with an unremarkable respiratory rate and oxygen saturation on room air.

Abdominal examination revealed mild diffuse tenderness, most marked in the epigastrium, without guarding, rigidity or rebound tenderness, and there were no clinical signs of peritonitis. Bowel sounds were audible. There was no seat‐belt sign, flank bruising or external abdominal wall injury. Examination of the chest, pelvis, and limbs was unremarkable, and there were no clinical features to suggest concomitant extra‐abdominal injury.

### 2.3 Timeline

The chronology of the episode of care is summarized in Table 1.

**TABLE 1:** Timeline of the episode of care.

Time point	Clinical events, findings, and interventions
Day 0—injury	Road traffic accident; blunt abdominal trauma sustained as a motorcycle pillion passenger following posterior impact.
Day 0—emergency department	Conscious and oriented. Persistent tachycardia despite intravenous fluid resuscitation. Mild abdominal tenderness without peritonism.
Day 0—laboratory testing	Hemoglobin 11.8 g/dL; serum amylase elevated at 597 U/L; total bilirubin 2.25 mg/dL; INR 1.32; arterial blood gas without acidosis.
Day 0—imaging	Contrast‐enhanced CT abdomen (portal venous phase): pancreatic tail lacerations with focal parenchymal discontinuity, peripancreatic collection 4.8 × 3.7 cm, mild haemoperitoneum. AAST Grade III injury suspected.
Day 0—operation	Exploratory laparotomy: complete transection of the pancreatic tail with main pancreatic duct disruption confirmed. Spleen‐preserving distal pancreatectomy performed; intra‐abdominal drain placed.
Postoperative Days 1–4	Haemodynamically stable. Minimal drain output. No fever, surgical site infection, respiratory compromise or sepsis. Pain mild and controlled with standard analgesia.
Postoperative Day 5	Drain removed; no biochemical, or clinical evidence of pancreatic fistula. Discharged in stable condition.
Week 2 (outpatient review)	Asymptomatic; wound healing satisfactorily; repeat serum amylase normalized.
Months 1–6 (surveillance)	Reviewed at regular intervals. Remains asymptomatic with no pancreatic fistula, intra‐abdominal collection, pseudocyst, or clinical features of endocrine/exocrine insufficiency (no formal dynamic function testing performed).

### 2.4 Diagnostic Assessment

#### 2.4.1 Diagnostic Testing

Initial laboratory investigations were as follows. Hemoglobin was 11.8 g/dL, total leukocyte count 9.6 × 10^3^/μL and platelet count 138 × 10^3^/μL. Renal function and electrolytes were within normal limits: blood urea nitrogen 11 mg/dL, creatinine 1.0 mg/dL, sodium 137 mEq/L, potassium 4.1 mEq/L, and chloride 99 mEq/L. Serum amylase was elevated at 597 U/L. Liver function tests showed a total bilirubin of 2.25 mg/dL, alanine aminotransferase (SGPT) 19 U/L, and alkaline phosphatase 86 U/L. The coagulation profile demonstrated an INR of 1.32, with prothrombin time and activated partial thromboplastin time within acceptable limits. Arterial blood gas analysis did not demonstrate acidosis.

Contrast‐enhanced computed tomography of the abdomen was performed in the portal venous phase. It demonstrated hypodense linear defects in the pancreatic tail consistent with parenchymal lacerations, together with focal discontinuity of the pancreatic parenchyma, raising strong suspicion for transection with involvement of the main pancreatic duct. Peripancreatic fat stranding and a localized fluid collection measuring approximately 4.8 × 3.7 cm were noted, consistent with hematoma. Mild haemoperitoneum was present. No active contrast extravasation was identified. The liver, spleen, kidneys, bowel loops and major abdominal vessels appeared unremarkable. These imaging findings raised suspicion of ductal disruption, and a provisional classification of suspected Grade III pancreatic injury according to the AAST Pancreatic Injury Scale was made [4]; ductal involvement could not be confirmed on CT alone. The contrast‐enhanced computed tomography appearances are shown in Figure 1.

**FIGURE 1:**
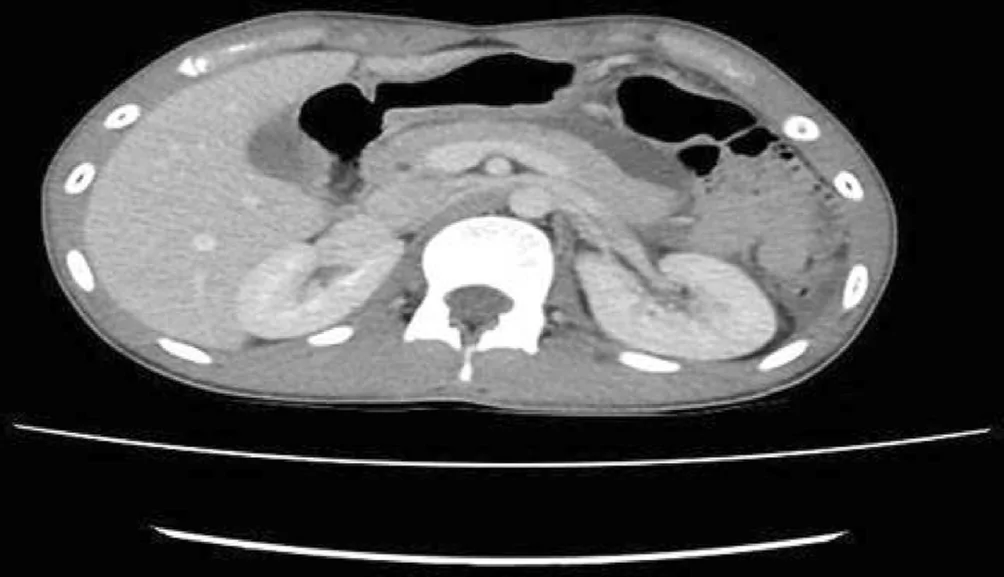
CT abdomen showing lacerations of the pancreatic tail along with discontinuity of parenchyma, raising suspicion of transection with main pancreatic duct involvement. A peripancreatic fluid collection is also observed. These indirect findings were suggestive of, but not diagnostic for, an AAST Grade III pancreatic injury, which was subsequently confirmed at laparotomy.

#### 2.4.2 Diagnostic Challenges

Several factors made the diagnosis challenging. The retroperitoneal position of the pancreas meant that the clinical examination remained relatively benign, with only mild tenderness and no peritonism, despite a complete ductal transection. Serum amylase is neither sensitive nor specific in the first hours after blunt abdominal trauma, so its elevation could not by itself establish or exclude ductal injury. Persistent tachycardia refractory to fluid resuscitation was therefore the principal clinical signal that a significant intra‐abdominal injury was present. Contrast‐enhanced computed tomography has variable sensitivity for direct visualization of the main pancreatic duct in the early phase, and the diagnosis rested on indirect signs, principally focal parenchymal discontinuity and a peripancreatic collection. Dedicated ductal imaging with magnetic resonance cholangiopancreatography or endoscopic retrograde cholangiopancreatography was not undertaken, as the patient required urgent operative exploration and these modalities were not appropriate within the available time frame.

#### 2.4.3 Diagnosis

The preoperative working diagnosis was an isolated AAST Grade III blunt pancreatic injury involving the pancreatic tail, with suspected disruption of the main pancreatic duct and an associated peripancreatic hematoma. Alternative diagnoses considered during the diagnostic work‐up included an AAST Grade I or II pancreatic contusion or laceration without ductal involvement, which would have permitted nonoperative management; concomitant solid organ injury to the liver, spleen or kidneys; hollow viscus or mesenteric injury; and a retroperitoneal hematoma of vascular origin. Each of these was considered unlikely on the basis of the computed tomography findings, which demonstrated normal appearances of the liver, spleen, kidneys, bowel loops and major abdominal vessels, and ductal transection, which could only be suspected on computed tomography, was confirmed at laparotomy.

#### 2.4.4 Prognosis

Grade III pancreatic injuries carry a recognized risk of pancreatic fistula, pseudocyst formation, intra‐abdominal abscess and, in the longer term, endocrine or exocrine insufficiency. In this patient, the anticipated prognosis was considered favorable given the isolated nature of the injury, the absence of shock or acidosis, the young age of the patient, and the short interval between injury and definitive resection.

### 2.5 Therapeutic Intervention

#### 2.5.1 Types of Intervention

Management comprised three components: initial resuscitative and supportive care in the emergency department; definitive operative management by exploratory laparotomy with spleen‐preserving distal pancreatectomy and intra‐abdominal drain placement; and perioperative pharmacological therapy including analgesia, antimicrobial prophylaxis, antiemetics, and venous thromboembolism prophylaxis.

The initial surgical plan was exploratory laparotomy with distal pancreatectomy, with or without splenectomy, depending on intraoperative findings and the assessment of the splenic vessels. On exploratory laparotomy, approximately 30 mL of haemoperitoneum was identified, and fat saponification involving the omentum and bowel loops was observed. Intraoperative findings confirmed complete transection of the pancreatic tail with disruption of the main pancreatic duct, consistent with the ductal injury suspected on preoperative computed tomography (Figure 2). The spleen and splenic vessels were intact, and splenic preservation was therefore achieved. A distal pancreatectomy was performed and an intra‐abdominal drain was placed. Splenectomy was not required.

**FIGURE 2:**
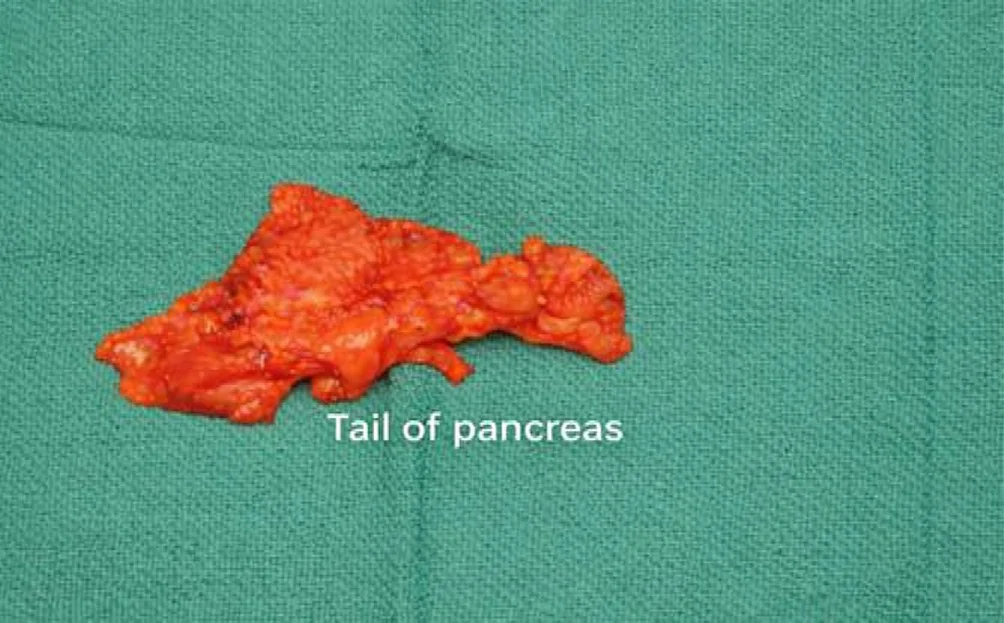
Intraoperative photograph showing complete transection of the pancreatic tail.

#### 2.5.2 Administration of Intervention

Definitive surgery was undertaken on the day of admission, without an intervening period of nonoperative observation. The procedure was performed under general anesthesia through an upper midline laparotomy incision. Approximately 30 mL of haemoperitoneum was evacuated at exploration, and intraoperative blood loss was otherwise minimal; no blood products were required. A single closed‐suction drain was placed in the pancreatic bed and left in situ for 5 days, being removed on the fifth postoperative day at the time of discharge.

Perioperative pharmacotherapy was administered in accordance with institutional protocol and comprised intravenous antimicrobial prophylaxis commenced at induction, multimodal analgesia with intravenous paracetamol and opioid rescue as required, and pharmacological venous thromboembolism prophylaxis started on the first postoperative day. Intravenous crystalloid was continued postoperatively and oral intake was reintroduced early and tolerated without difficulty. Somatostatin analog therapy was not administered. The total length of hospital stay was 5 days.

#### 2.5.3 Changes in Intervention

One planned intervention was modified intraoperatively. Splenectomy had been anticipated as a possible requirement at the time of consent and operative planning, since distal pancreatectomy for tail injury frequently necessitates removal of the spleen when the splenic vessels are involved. Direct inspection at laparotomy demonstrated that the splenic artery and vein were intact and that the spleen was viable and uninjured. The procedure was therefore modified to a spleen‐preserving distal pancreatectomy, with the rationale of avoiding the lifelong risk of overwhelming postsplenectomy infection and the associated need for vaccination and prophylaxis in an otherwise healthy adolescent. No other changes to the planned therapeutic strategy were required.

### 2.6 Follow‐Up and Outcomes

#### 2.6.1 Clinician‐ and Patient‐Assessed Outcomes

The postoperative course was uneventful. The patient remained haemodynamically stable throughout, with no clinician‐observed evidence of surgical site infection, acute respiratory distress syndrome or sepsis. Drain output was minimal, and there was no clinical or biochemical evidence of pancreatic fistula. The drain was removed on the day of discharge and the patient was discharged on the fifth postoperative day in stable condition. From the patient's perspective, postoperative pain was mild and adequately controlled with standard analgesia, and he reported no abdominal complaints at outpatient review.

#### 2.6.2 Follow‐Up Diagnostic and Other Test Results

The patient was reviewed in the outpatient clinic 2 weeks after discharge. He was clinically stable and asymptomatic, and the surgical wound was healing satisfactorily. Repeat serum amylase obtained at this visit had normalized. He was subsequently followed at regular intervals over a period of 6 months. At the most recent review he remained clinically stable, with no clinical or biochemical evidence of post‐traumatic pancreatic fistula, intra‐abdominal collection or pseudocyst formation, and no clinical features suggestive of endocrine or exocrine insufficiency. Surveillance was clinical and biochemical; no formal dynamic testing of pancreatic endocrine or exocrine function was undertaken, and subclinical insufficiency therefore cannot be excluded.

#### 2.6.3 Intervention Adherence and Tolerability

Adherence was assessed by attendance at scheduled outpatient appointments and by clinical review of symptoms at each visit. The patient attended all scheduled follow‐up appointments over the six‐month surveillance period and continues to undergo routine clinical surveillance in accordance with standard post‐pancreatectomy follow‐up protocols. The operative and pharmacological interventions were well tolerated; no prescribed medication was discontinued early and no intolerance to analgesia or antimicrobial therapy was reported.

#### 2.6.4 Adverse and Unanticipated Events

No adverse or unanticipated events occurred during the inpatient stay or over the six‐month follow‐up period. Specifically, there were no procedure‐related complications, no postoperative pancreatic fistula as defined by the International Study Group of Pancreatic Surgery criteria, no pseudocyst, no intra‐abdominal collection requiring drainage, no wound complication, and no readmission. The intraoperative conversion from a planned distal pancreatectomy with possible splenectomy to a spleen‐preserving procedure was a favorable deviation from the operative plan rather than an adverse event.

### 2.7 Patient Perspective

The patient reported that he had not initially appreciated the severity of his injury, as his abdominal pain was mild and he felt otherwise well after the accident. He described being reassured by the explanation that the scan had shown a significant injury to the pancreas requiring an operation. He found the hospital stay shorter than he had expected and reported that his postoperative pain was mild and well controlled with the analgesia provided. By the time of his outpatient review, he had resumed his normal daily activities and studies without limitation and reported no abdominal symptoms. He expressed satisfaction with the care received and stated that his main remaining concern was whether the removal of part of the pancreas would affect his health in the longer term, for which he was reassured and offered continued follow‐up.

### 2.8 Informed Consent

Written informed consent was obtained from the patient for publication of this case report and the accompanying images. A copy of the signed consent form is available for review by the Editor‐in‐Chief of this journal on request.

## 3 Discussion

Pancreatic trauma is uncommon, particularly when occurring in isolation. Blunt abdominal trauma, especially due to road traffic accidents, is the most frequent mechanism and typically results from compression of the pancreas against the vertebral column [5].

A major challenge in pancreatic trauma lies in the early identification of main pancreatic duct injury, which is the key determinant of management. While contrast‐enhanced computed tomography is the imaging modality of choice in hemodynamically stable patients, its sensitivity in detecting ductal injury has been reported to be variable, particularly in the early phase [6].

In this case, CT imaging demonstrated parenchymal discontinuity and associated peripancreatic fluid collection, raising early suspicion of ductal involvement, which was subsequently confirmed intraoperatively. This highlights the importance of recognizing subtle radiological signs that may indicate ductal disruption.

These CT findings are indirect and were interpreted as suspicious for, rather than diagnostic of, main pancreatic duct injury. Dedicated ductal imaging with magnetic resonance cholangiopancreatography or endoscopic retrograde cholangiopancreatography allows direct assessment of ductal integrity and is recommended in haemodynamically stable patients when ductal injury is suspected and the clinical situation permits [1, 6, 7]. In this patient, ductal transection was confirmed only at laparotomy, and concordance between computed tomography and operative findings in a single case cannot be taken as evidence of the diagnostic reliability of computed tomography for ductal injury.

According to the AAST Pancreatic Injury Scale, Grade III injuries involve distal pancreatic transection with ductal disruption and typically require operative management [1, 4]. In this patient, preoperative suspicion of ductal injury, rather than a confirmed ductal diagnosis, was considered sufficient to proceed with timely surgical planning and definitive management.

Distal pancreatectomy, with or without splenectomy, remains the standard treatment for injuries involving the body and tail of the pancreas [7]. In this case, splenic preservation was successfully achieved [8].

We acknowledge that Grade III pancreatic injury with main duct disruption is a well‐characterized entity and that the operative management described here is consistent with current WSES–AAST recommendations [1]. The contribution of this report is therefore educational rather than novel. First, it documents the operative correlation of each indirect CT sign on which preoperative suspicion rested, which may assist clinicians interpreting early imaging in stable patients. Second, it illustrates that, when magnetic resonance cholangiopancreatography or endoscopic retrograde cholangiopancreatography cannot be obtained without delaying surgery, a well‐founded CT suspicion of distal ductal injury may support operative planning, while recognizing that this approach carries a risk of operating on injuries that dedicated ductal imaging would have shown to be of lower grade. Third, it reinforces that persistent tachycardia refractory to fluid resuscitation, in the absence of external signs or peritonism, may be the principal early clinical clue to isolated pancreatic injury.

Prompt surgical intervention following early CT‐based suspicion of ductal injury may have contributed to the absence of postoperative complications, including pancreatic fistula, pseudocyst formation, and intra‐abdominal sepsis, although no causal inference can be drawn from a single case.

### 3.1 Strengths and Limitations

The principal strength of this report is the direct correlation between a specific preoperative radiological sign, focal parenchymal discontinuity with a peripancreatic collection, and the intraoperative confirmation of complete ductal transection, which provides a clear illustration of how ductal injury can be suspected on contrast‐enhanced computed tomography when direct ductal visualization is not available. The report also documents a complete six‐month follow‐up period with an explicit account of the complications that were sought and not found, and it documents the intraoperative correlation of every radiological feature on which the preoperative suspicion rested.

Several limitations should be acknowledged. As a single case report, these observations cannot be generalized and no inference can be drawn about the sensitivity or specificity of the computed tomography findings described. Ductal disruption was not confirmed preoperatively by magnetic resonance cholangiopancreatography or endoscopic retrograde cholangiopancreatography, so the computed tomography findings could be verified only retrospectively against the operative findings, and the preoperative diagnosis should be regarded as a suspected rather than confirmed ductal injury. Follow‐up was clinical and biochemical, and as no formal dynamic testing of pancreatic function was performed, subclinical endocrine or exocrine insufficiency cannot be excluded on clinical grounds alone. In addition, 6 months is a relatively short interval in which to exclude late sequelae such as pseudocyst formation or the delayed onset of pancreatic insufficiency, and longer‐term surveillance is ongoing. Finally, the clinical scenario and management described are consistent with established guideline‐based care, and this report does not describe a novel entity or therapeutic approach.

## 4 Conclusion

Isolated pancreatic injury following blunt abdominal trauma is rare and diagnostically challenging. Early suspicion of main pancreatic duct involvement on contrast‐enhanced CT is important in guiding management. This case highlights that careful interpretation of imaging findings can raise a well‐founded preoperative suspicion of ductal injury that, in a hemodynamically stable patient, may support timely surgical intervention, while definitive confirmation of ductal injury requires dedicated ductal imaging or operative assessment.

## 5 Learning Points

- Early CT findings such as parenchymal discontinuity and peripancreatic fluid should raise suspicion of main pancreatic duct injury, although they are indirect and not definitive.
- Preoperative CT suspicion of ductal involvement can support timely surgical planning when dedicated ductal imaging cannot be obtained without delay.
- Isolated pancreatic trauma can occur in the absence of other intra‐abdominal injuries.
- Persistent tachycardia despite fluid resuscitation may be the principal early clinical sign of isolated pancreatic injury when abdominal examination is relatively benign.
- Prompt operative management of Grade III injuries was associated with a favorable outcome in this case.

## Author Contributions

**Syeda Sarah Kazmi:** conceptualization, writing – original draft, writing – review and editing, visualization. **Syeda Zubaria Qamar:** writing – original draft, writing – review and editing. **Ahmed Ali:** writing – original draft, writing – review and editing. **Manahil Sohail Fatani:** writing – original draft, writing – review and editing. **Syed Ali Haider:** writing – original draft, writing – review and editing, visualization. **Muhammad Farhan:** writing – original draft, writing – review and editing. **Ahmed Asad Raza:** writing – original draft, writing – review and editing, supervision, methodology, software. **Abedin Samadi:** writing – original draft, writing – review and editing.

## Funding

The authors have nothing to report.

## Ethics Statement

Institutional review board approval is not required for the publication of a single de‐identified case report at our institution. Written informed consent was obtained from the patient, and the work was conducted in accordance with the principles of the Declaration of Helsinki.

## Consent

Written informed consent was obtained from the patient for publication of this case report and any accompanying images. A copy of the written consent is available for review by the Editor‐in‐Chief of this journal on request.

## Conflicts of Interest

The authors declare no conflicts of interest.

## Data Availability

All data relevant to this case report are included within the article. Further de‐identified information is available from the corresponding author on reasonable request.
